# Clinical efficacy and problems with CT lymphography in identifying the sentinel node in breast cancer

**DOI:** 10.1186/1477-7819-6-57

**Published:** 2008-06-12

**Authors:** Masako Takahashi, Mitsunori Sasa, Chieko Hirose, Sonoka Hisaoka, Masako Taki, Toshiyuki Hirose, Yoshimi Bando

**Affiliations:** 1 Department of Radiology, Tokushima Breast Care Clinic, 4-7-7, Nakashimada-Cho, Tokushima, 770-0052, Japan; 2 Department of Surgery, Tokushima Breast Care Clinic, 4-7-7, Nakashimada-Cho, Tokushima, 770-0052, Japan; 3 Department of Radiology, National Higashi Tokushima Hospital, 1-1, Ohmukai-kita, Ootera, Itano, Tokushima, 779-0193, Japan; 4 Department of Radiology, Tokushima Prefecture Hospital, 1-10-3, Kuramoto-cho, Tokushima, 770-8539, Japan; 5 Department of Surgery, National Higashi Tokushima Hospital, 1-1, Ohmukai-kita, Ootera, Itano, Tokushima, 779-0193, Japan; 6 Department of Molecular and Environmental Pathology, Institute of Health Biosciences, The University of Tokushima Graduate School, 3-18-15, Kuramoto-Cho, Tokushima, 770-8509, Japan

## Abstract

**Background:**

Combining a radioisotope with a dye-guided method is the best method for identification of the sentinel lymph nodes (SNs) in breast cancer. However, some institutions are limited to use of a dye-guided method alone. Recently, computed tomographic lymphography (CTLG) employing a nonionic contrast medium has achieved SN identification.

**Patients and methods:**

218 patients with primary breast cancer and no clinical evidence of lymph node metastasis were studied. SN identification was performed by CTLG and a dye-guided method. The SN identification rate was analyzed for correlations with the clinicopathological findings.

**Results:**

The SN identification rates were 96% with CTLG, 92% with the dye-guided method and 99% with both methods combined. The identification rates with CTLG and the combined method were significantly lower in node-positive patients compared to node-negative patients, and significantly lower with the combined method in vascular invasion-positive patients compared to negative patients. In addition, the SN identification rate with the dye-guided method was significantly lower in patients with a body mass index (BMI) of ≥ 25, whereas the BMI did not affect the identification rate with CTLG or the combined method. Multiple SNs were detected in approximately 20% of the patients.

**Conclusion:**

Combined performance of CTLG and a dye-guided method enables identification of SNs prior to breast cancer surgery. That SN identification is easier compared with by the dye-guided method alone, and the identification rate is improved compared with either method alone. The combination of methods was especially useful in obese patients. For patients with multiple SNs, the combination has the further advantage of enabling accurate SN biopsy. CTLG may yield false-negative findings in node-positive patients and patients with lymph vessel obstruction.

## Background

Sentinel node biopsy (SNB) has become a standard surgical procedure for patients with early-stage breast cancer [1,2]. The sentinel nodes (SN) can be identified by a double-mapping procedure based on a gamma probe-guided method and a dye-guided method using a radioisotope (RI), or by a triple-mapping procedure that includes lymphoscintigraphy and is even more effective [1-3]. However, the RI method can be performed only at institutions that are trained and licensed to use RI, and other institutions must rely on dye methods alone for SN identification [4-8] On the other hand, for obtaining images of the lymph vessels and nodes, indirect lymphography seems to be a more convenient than direct intralymphatic administration of a contrast medium. Several studies of indirect lymphography were reported in the 1980s [9-12], and Suga *et al*., reported successfully identifying SN by three-dimensional computed tomographic lymphography (CTLG) using a nonionic contrast medium [6.7]. We have also been identifying SN in breast cancer patients by CTLG and a dye-guided method since February 2003 as a clinical trial [5]. Here, we report our findings to date regarding the clinical efficacy and problems associated with SN identification by the combination of CTLG and a dye-guided method.

## Patients and methods

All studies in this paper were approved by the ethics committee of National Higashi-Tokushima Hospital. After presenting a detailed explanation of this clinical trial, written informed consent was obtained from all patients.

### Patients

During the period from February 2003 through March 2007, 218 Japanese patients with T1N0M0 or T2N0M0 primary breast cancer were treated at the Tokushima Breast Care Clinic. SN identification was performed by combined application of CTLG and a dye-guided method. In two of the patients the CTLG was performed before and after excisional biopsy, and in one patient with bilateral disease CTLG was performed on both sides. Thus, CTLG was performed 221 times in total, while the dye-guided method was performed a total of 219 times. In principle, backup dissection was performed for patients found to be SN metastasis-positive provided that informed consent was granted. For metastasis-negative patients, axillary dissection was omitted on the basis of informed consent.

### Methods

CTLG was performed as previously described [5]. Briefly, the CT apparatus was a high-speed FX/I single-detector helical CT scanner (GE Yokogawa Medical Systems, Tokyo, Japan). One mL of iopamidol (Iopamiron 300; 300 mgI/ml, Schering, Osaka, Japan) was injected subcutaneously to the areola or both subcutaneously to the areola and subcutaneously directly above the tumor, and the SN was attempted to be identified by CT performed 1 min later. In the case that the enhancement of the lymph vessels and lymph nodes was poor, the site(s) where the contrast medium had been injected was massaged, and after 3–5 min the CT was repeated. SNs were predicted from CT images by identification of enhanced lymph vessels and/or lymph nodes and assessment of the CT values. The CT values of lymph nodes were measured at their maximally enhanced point. If an SN(s) was identified, a mark was made on the skin immediately above the SN. The dye-guided method was also performed as previously described [5]. Briefly, 2~3 mL of indigo carmine blue was injected subcutaneously to the areola, followed by massage of the injection site for a few minutes. Just before performing an operation, we used ultrasonography to confirm the location of SN(s) that had been identified by CTLG. Five to 15 min later a skin incision was made at the site(s) that were marked in the CTLG, and any blue-stained lymph vessels and lymph nodes were identified. If no SN(s) was identified by either the dye-guided method or CTLG, axillary lymph node dissection was performed.

### Definition of SNs

*CTLG*: A lymph node was defined as an SN if visual inspection or the CT value (the increase of Hounsfield units after subcutaneous injection) confirmed it to be enhanced or if it was confirmed to connect to an enhanced lymph vessel.

*Dye-guided method*: A lymph node was defined as an SN if it was dyed blue or if it was confirmed to connect to a blue lymph vessel.

### SN identification rates and the clinicopathological findings

The results were analyzed in relation to the success/failure of SN identification and various clinicopathological parameters, including the patient age, menopausal status, body mass index (BMI), tumor diameter, presence/absence of excisional biopsy, histological type, presence/absence of lymph node metastasis and presence/absence of vascular invasion.

### Statistical analysis

The relationships between the success/failure of SN identification and the clinicopathological findings were analyzed for statistical significance using the chi-square test. A p value of <0.05 was considered to indicate statistical significance.

## Results

### SN identification rates by CTLG and the dye-guided method

Identification of the SN(s) was achieved in 212 (96%) of the total 221 performances of CTLG. The number of detected SNs ranged from 1 to 3, with a mean of 1.2 SNs. With the dye-guided method the identification rate was 92% (202/219 tests). The combined identification rate was 99% (Table 1). With CTLG, both the lymph vessels and lymph nodes were clearly enhanced and the SNs could be identified in 189 patients (86%). In 5 patients (2%) the lymph vessels were not enhanced but the lymph nodes were, and the SNs could be identified. In 7 other patients (3%), the lymph vessels were enhanced while the lymph nodes were not clearly enhanced, but the SNs could be identified from the CT value. In 5 patients (2%) neither the lymph vessels nor the lymph nodes were enhanced, but the SNs could be identified from the CT value. In 6 patients (3%) the lymph vessels were enhanced but the lymph nodes were not, but the SNs could be identified on the basis of confirmation of their connection to the enhanced lymph vessels. Finally, in 9 patients (4%) neither the lymph vessels nor the lymph nodes were enhanced, and SNs could not be demonstrated (Table 2).

**Table 1:** Identification rates of lymph vessels and sentinel lymph nodes

	**Dye**	**CTLG**	**Combination**
**Lymph vessels**	212 (97%)	202 (91%)	215 (98%)
**Sentinel lymph node**	202 (92%)	212 (96%)	216 (99%)

Dye : Dye-guided method

CTLG : CT Lymphography

Combination: Combination of CTLG and Dye

**Table 2:** Findings of CT lymphography n = 221

**Lymph vessels**	**Nodes**	
**Enhanced**	Clearly enhanced	189 (86%)
**Unenhanced**	Clearly enhanced	5 (2%)
**Enhanced**	Assessment of CT values	7(3%)
**Unenhanced**	Assessment of CT values	5(2%)
**Enhanced**	Unenhanced	6(3%)
**unenhanced**	unenhanced	9(4%)

### SN identification rates and the clinicopathological findings

Analysis of the relationships between the SN identification rates and the clinicopathological findings showed that the SN identification rates with CTLG, the dye-guided method and the combined method showed no differences as a function of the age, menopausal status, tumor diameter or histopathological type. However, with the dye-guided method the SN identification rate was significantly lower in patients with a BMI of 25 or higher, whereas with CTLG and the combined method the SN identification rate was not influenced by the BMI. With CTLG and the combined method, the SN identification rate was significantly lower in the node-positive patients, and with the combined method it was significantly lower in patients with vascular invasion. In addition, in the patients who had undergone lateral-upper region excisional biopsy the SN identification rate with CTLG was lower than in the patients who had undergone excisional biopsy in a region other than the lateral-upper region, although the difference did not reach statistical significance. Moreover, with the dye-guided method the SN identification rate was lower in the patients who had undergone excisional biopsy, regardless of the region, compared with the patients who had not undergone excisional biopsy (Table 3).

**Table 3:** Sentinel node (s) identification rate and clinicopathological findings (all cases) (Dye n = 219, CTLG n = 221, Combination n = 219)

	**Dye (+)(n = 202)**	**CTLG (+)(n = 212)**	**Combination (+)(n = 216)**
**Age, years**			
**≦35**	6(100%)	6(100%)	6(100%)
**36~50**	87(90%)	92(95%)	96(98%)
**≧51**	109(94%)	114(97%)	114(98%)
**Menopausal state**			
**Pre**	103(92%)	108(96%)	105(98%)
**Post**	99(93%)	104(95%)	111(99%)
**BMI**			
**<25**	160(95%) #	164(96%)	166(98%)
**≧25**	42(84%)	48(96%)	50(100%)
**Tumor size, cm**			
**≦2.0**	183(92%)	193(96%)	197(99%)
**≧2.1**	19(100%)	19(100%)	19(100%)
With excisionary biopsy*			
**In lateral-upper region**	17(89%)	18(95%)	19(100%)
**In other regions**	46(88%)	52(100%)	52(100%)
**Histological type**			
**IIa1**	71(88%)	80(99%)	80(99%)
**IIa2**	22(100%)	23(100%)	22(100%)
**IIa3**	64(96%)	63(94%)	65(97%)
**IIb3**	8(89%)	8(89%)	9(100%)
**DCIS**	13(100%)	12(92%)	13(100%)
**Others**	24(89%)	26(93%)	27(100%)
**Nodal status**			
**n(-)**	177(93%)	189(98%) #	191(100%) #
**n(+)**	25(89%)	23(82%)	25(89%)
**Vascular invasion**			
**v(-)**	160(91%)	172(97%)	174(99%) #
**v(+)**	42(95%)	40(91%)	42(95%)

BMI : Body mass index

Dye : Dye-guided method

CTLG : CT Lymphography

Combination: Combination of CTLG and Dye

#; P<0.05

*; cases with excisional biopsy (n=71)

### Size of the metastasis and the SN identification rate in node-positive patients

The patients found to be positive for metastasis were classified and analyzed on the basis of the size of the metastasis: <0.2 mm, 0.2~2 mm and >2 mm. The results showed that the SN identification rate by both CTLG and the dye-guided method decreased as the size of the metastasis increased, although the difference did not reach statistical significance (Table 4).

**Table 4:** Size of the metastatic lesion in the node positive cases n = 28

**Metastatic lesion size**	**Dye(+)**	**CTLG(+)**
**Isolated tumor cells(<0.2 mm)**	2(100%)	2(100%)
**Micrometastasis(0.2~2 mm)**	10(91%)	9(82%)
**Macrometastasis(≧2 mm)**	13(87%)	12(80%)

Dye: Dye-guided method, CTLG: CT Lymphography

### Number of lymph vessels leading to SNs and the number of SNs

In this study, we were able to analyze the data on the lymph vessels leading to SNs and the number of SNs of 200 patients. A single route with a single SN was the most common pattern, seen in 68% of the patients. Multiple routes with a single SN were detected in 4% of the patients, while a single route with multiple SNs was seen in 10% and multiple routes with multiple SNs were seen in 8%. Overall, multiple SNs were identified in 18% of the patients (Table 5).

**Table 5:** Number of lymph vessels leading to Sentinel nodes and the number of Sentinel nodes

Single rout and Single node	151(68%)
Multi routs and single node	8(4%)
Single rout and multi nodes	23(10%)
Multi routs and multi nodes	18(8%)

## Discussion

In breast cancer surgery, dissection of the axillary lymph nodes is considered effective for the objectives of performing staging and achieving local control [1,13]. Therefore, in recent years SNB has become a standard procedure for patients with no metastasis of the axillary lymph nodes since it avoids unnecessary dissection [1,2]. Combined use of an RI (gamma probe-guided method and lymphoscintigraphy) and a dye is currently considered to be more efficient for identification of the SN(s) than single use of either of these methods [1-3]. In Japan, there are many institutions that do not have the necessary facilities for using RIs and thus must use the dye-guided method alone to identify SNs [4,5]. On the other hand, Suga et al. proposed using a method called CTLG, which employs a nonionic contrast medium [6,7], and we have applied that method for SN identification since February 2003 [5]. In this paper we have reported our findings regarding the usefulness and problems associated with combined application of CTLG and the dye-guided method for SN identification.

Our experimental subjects were 219 Japanese patients with primary breast cancer that was thought to be clinically free of axillary lymph node metastasis. CTLG and the dye-guided method were employed in an attempt to identify the SN(s) in each of the patients. The SN identification rates were 96% with CTLG alone, 92% with the dye-guided method alone and 99% when the findings with the two methods were combined. Those results are good when compared with the published data for combined use of an RI and the dye-guided method [2,3]. In particular, our SN identification rate with the dye-guided method is better than the rates that have been reported to date [2,3,14]. We think that this is because the location of the SN had already been determined by the CTLG, making it easy to identify the lymph vessels and lymph nodes. The breakdown of the identification by CTLG showed clear enhancing of the lymph nodes in 88% of the patients, while in 5% of the patients identification of the SN was possible on the basis of the CT value in spite of the fact that the lymph nodes were not clearly enhanced. Because the observations were macroscopic, when the dye-guided method was used alone, SN identification was difficult unless the lymph node was stained to a sufficient degree. With CTLG, on the other hand, even if the enhancement is not very striking it can be surmised that the SN identification rate will be improved since the CT value can be taken into consideration.

In 14 patients, the SN was identified by CTLG, but not by the dye-guided method. The location of the SN was confirmed by ultrasonography just before the operation in all of these patients. In these 14 patients, not only the SN but also neighboring nodes were sampled at the same time. Therefore, it seemed that the SN biopsies had been performed accurately.

The sensitivity of CTLG could not be investigated in our patient population because backup axillary dissection was not performed for patients who were metastasis-negative in the SNB. In addition, none of the patients experienced postoperative recurrence in the axillary lymph nodes, but that does not serve as a basis for claiming that there were few false-negatives [1]. However, Tangoku *et al*., reported the sensitivity of CTLG to be 98%, and for that reason it can be thought that the sensitivity of CTLG is not inferior to that of the RI method [15].

With regard to correlations between the clinicopathological findings and the SN identification rate, the patient age, the location of the tumor, whether or not surgical biopsy was performed, the tumor size and the presence/absence of lymph node metastasis have been reported to influence identification of the SN [1,16-18]. In our patient series, the results showed no differences as a function of the age, menopausal status, tumor diameter or histopathological type. However, with the dye-guided method the SN identification rate was significantly lower in patients with a BMI of 25 or higher, whereas with CTLG and the combined method the SN identification rate was not influenced by the BMI. As the reason for this difference it is noted that with the dye-guided method it can be difficult to discern staining of lymph vessels and nodes with the naked eye if considerable subcutaneous fat is present, whereas with CTLG the subcutaneous fat plays no role since the observation is done by CT. In addition to the BMI, discrepancies between the SN identification rates with these three methods were observed as a function of the presence/absence of lymph node metastasis, the site of excisional biopsy (i.e., the lateral upper region and regions other than the lateral upper region), and the presence/absence of vascular invasion. Especially, the identification rates with CTLG and the combined method were significantly lower in node-positive patients compared to node-negative patients, and significantly lower with the combined method in vascular invasion-positive patients compared to negative patients. The size of injected particles, the injected volume and the injection site have been reported as examination factors that can influence SN identification [2]. The nonionic contrast media that are used in CTLG have a larger particle size than dyes [19]. For that reason it can be hypothesized that, in patients with lymph node metastasis or vascular invasion and in patients who had undergone lateral upper region excisional biopsy, who can be predicted to have lymph vessel occlusion, the movement of a nonionic contrast medium would be impeded compared with that of a dye, thus resulting in a lower SN identification rate. On the other hand, the SN identification rate with the dye-guided method was lower in the patients with excisional biopsy in any region than in patients without excisional biopsy. It can be hypothesized that this is probably because excisional biopsy leads to edema of the connective tissue, which makes it difficult to distinguish the dye.

We also investigated the number of SNs and the number of lymph vessels leading to them [20-22]. These evaluations are difficult to achieve by the gamma probe-guided method and the dye-guided method, but CTLG permits detailed investigation. Our results showed that there was a single route leading to a single SN in 68% of the patients, multiple routes leading to a single SN in 4%, a single route leading to multiple SNs in 10% and multiple routes leading to multiple SNs in 8%. These results are in agreement with those reported by Tangoku *et al*., [23]. Thus, approximately 18% of the patients in our series had multiple SNs, and it can be hypothesized that it would be difficult to biopsy all of them if only the dye-guided method were employed. We think that combined use of CTLG with the dye-guided method would permit accurate biopsy, and for that reason we anticipate that combined performance of CTLG will prove useful.

CTLG is a diagnostic test that is performed prior to surgery for breast cancer. Studies are warranted to determine whether the CT findings or performance of fine-needle aspiration cytology of the SN will make it possible to achieve diagnosis of SN metastasis preoperatively and then decide whether or not SNB should be performed [24]. Such diagnosis leading to avoidance of unnecessary surgical procedures would represent a great clinical advantage by reducing the burden on the patient. Moreover, in the future it will be necessary to compare the usefulness of CTLG with the RI method.

## Conclusion

Combined performance of CTLG with the dye-guided method permits better elucidation of the location of the SN(s) in breast cancer. This makes it easier to identify the SN(s) and results in a higher SN identification rate compared with application of the dye-guided method alone. Combination of CTLG and the dye-guided method was especially useful in obese patients. In addition, in patients with multiple SNs, it is advantageous to be able to perform SNB accurately. However, in patients with occlusion of the lymph vessels due to lymph node metastasis, having undergone lateral upper region lumpectomy or the presence of vascular invasion, there is a possibility that false-negative diagnostic results will be generated with CTLG.

## Abbreviations

SN: sentinel node; SNB: sentinel node biopsy; CTLG: computed tomographic lymphography; BMI: body mass index; RI: radioisotope.

## Competing interests

The authors declare that they have no competing interests.

## Authors' contributions

MS initiated and co-wrote the paper with MT and CH, YB examined surgical specimen, MS, SH, MT and TH took part in the care of patients and helped in preparation of the manuscript. All authors read and approved the manuscript.
